# The Clinical Impact of Sarcopenia and Delirium in Hospitalized Elderly Patients: An Analysis Using Muscle Ultrasound

**DOI:** 10.1002/jcsm.70202

**Published:** 2026-01-28

**Authors:** Thomas Fraccalini, Laura Santos Ribeiro, Andrea Trogolo, Beatrice Tarozzo, Valerio Piras, Julia Michelin Vecchini, Rouslan Senkeev, Oksana Sukhova, Luciano Cardinale, Giuseppe Maina, Salvatore Di Gioia, Davide Minniti, Truce Massimiliano, Binello Elisa, Finiguerra Ivana, Roberta Vacchelli, Fustamante Elda, Romano Gianfranco, Turja Reald, Valerio Ricci, Alessandro Maraschi, Thomas Roberts, Giovanni Volpicelli

**Affiliations:** ^1^ Department of Medical and Oncology, Division of Geriatrics Medicine San Luigi Gonzaga Hospital Turin Italy; ^2^ Department of Clinical and Biological Sciences, Medicine and Surgery University of Turin Turin Italy; ^3^ Division of Geriatrics Medicine University of Genoa Genoa Italy; ^4^ University Guglielmo Marconi Rome Italy; ^5^ Independent General Practitioner and Emergency Physician Turin Italy; ^6^ Radiology Unit Turin Italy; ^7^ Department of Psychiatry San Luigi Gonzaga University Hospital Turin Italy; ^8^ Department of Neurosciences “Rita Levi Montalcini”, University of Turin Turin Italy; ^9^ San Luigi Gonzaga University Hospital Turin Italy; ^10^ Department of Thoracic Surgery University College London Hospitals, University College Hospital at Westmoreland Street London UK; ^11^ University College London Medical School University College London London UK; ^12^ Department of Medical and Surgical Sciences, Division of Emergency Medicine Magna Graecia of Catanzaro Catanzaro Italy

**Keywords:** delirium, elderly, geriatric patients, muscle ultrasound, sarcopenia, Ultrasound Sarcopenia Index, vastus lateralis

## Abstract

**Background:**

Sarcopenia and delirium are two highly prevalent clinical syndromes among hospitalized elderly patients, both independently associated with adverse outcomes such as increased risk of falls, disability and mortality. Although a correlation between sarcopenia and delirium has been previously reported, past studies have often relied on less reliable surrogate markers for sarcopenia, leading to potential inaccuracies in diagnosis and assessment. This study aims to address these limitations by utilizing a more precise and reliable diagnostic tool for sarcopenia, specifically, muscle ultrasound (US) to measure the quadriceps femoris muscle and its pennation angle, to accurately evaluate the correlation between sarcopenia and delirium in acutely admitted geriatric patients.

**Methods:**

We used muscle US to measure sarcopenia with reliable markers, specifically the Ultrasound Sarcopenia Index (USSI). This index evaluates the ratio between vastus lateralis thickness and fascicle length (L/f), offering a detailed view of muscle structure that is not affected by sex or body size.

**Results:**

In 194 patients (mean age 86.5 years; 46.4% women; 81.4% with delirium), USSI correlated with MMSE (*r* = −0.619, *p* < 0.001), 4AT (*r* = 0.844, *p* < 0.001), ADL/IADL (*r* ≈ −0.32, *p* < 0.001), BMI, grip strength and muscle thickness (all *p* < 0.001). Women had higher USSI and worse cognitive/frailty scores; patterns were consistent across sexes.

**Conclusion:**

Our findings are expected to support the implementation of more targeted assessments and interventions, emphasizing the crucial role of accurate sarcopenia diagnosis in improving outcomes for elderly patients and highlighting its interconnectedness with delirium in comprehensive geriatric care.

## 1 Introduction

Sarcopenia and delirium are two highly prevalent clinical conditions with a significant impact on the health of elderly individuals, particularly those hospitalized [1]. Both are independently associated with adverse outcomes, including increased risk of falls, disability and mortality [2].

Sarcopenia is a progressive, age‐related musculoskeletal disease characterized by a reduction in muscle mass, strength and physical performance [3]. It represents a significant independent risk factor for a range of complications, including falls, disability, postoperative morbidity and mortality [4, 5, 6]. A definitive diagnosis of sarcopenia typically requires the presence of at least two of the following three criteria: (1) low skeletal muscle mass, (2) reduced muscle strength and (3) poor physical performance [7]. Though a natural part of ageing, sarcopenia is often exacerbated by factors common in the elderly, including malnutrition, physical inactivity, comorbidities and iatrogenic events [8]. Among elderly inpatients, it is often present at admission and significantly affects both short‐term outcomes and long‐term recovery [9]. Therefore, early identification is crucial, and sarcopenia assessment should be considered a fundamental component of comprehensive care for high‐risk, frail elderly patients [10].

Traditional diagnostic methods such as magnetic resonance imaging (MRI) and dual‐energy x‐ray absorptiometry (DXA) are often costly, bulky and impractical for routine clinical use [11, 12, 13]. These limitations hinder their widespread application. With this in mind, muscle ultrasound (US) has emerged as an extremely valuable and innovative alternative diagnostic tool [14, 15]. Unlike conventional methods, US is portable, low‐cost and non‐ionizing. Besides US being able to measure the cross‐sectional area and volume of muscle, there is the additional advantage of detecting subtle yet clinically relevant changes in muscle architecture, measured in the fascicle length (FL) and the pennation angle (θ) [15, 16].

These architectural changes serve as key indicators of sarcopenia and can be accurately quantified using US‐based approaches like the Ultrasound Sarcopenia Score Index (USSI). By assessing the ratio between vastus lateralis thickness and fascicle length, the USSI provides a reliable and versatile marker—with the added advantage of being independent of sex and body size [15, 16, 17].

Delirium, also known as acute confusional state, is a clinical syndrome characterized by an abrupt and fluctuating alteration in consciousness, perception and attention [18, 19]. It is frequently underdiagnosed and misattributed to normal ageing. However, the onset of delirium during hospitalization significantly increases the risk of short‐term mortality, the likelihood of institutionalization and the development of future physical disability [18, 19, 20, 21]. Moreover, it also exposes patients to a higher risk of subsequent neurodegenerative disorders, such as Alzheimer's disease [18]. Diagnosis can be particularly challenging in cases of hypoactive delirium, where symptoms like lethargy and reduced activity obscure clinical detection [19]. Contributing factors to delirium are multifaceted and include advanced age, pre‐existing dementia, depression, sensory impairments, psychotropic drug use, invasive procedures and metabolic or thermoregulatory disturbances [22, 23].

Sarcopenia and delirium are not merely coexisting conditions; they are intrinsically correlated [24, 25]. Previous studies have demonstrated an independent association between sarcopenia and the development of delirium in acutely ill hospitalized geriatric patients [2]. For instance, Bellelli et al. reported that sarcopenia (as defined by the criteria of the European Working Group on Sarcopenia in Older People) was independently associated with the onset of delirium in this population [26]. However, previous sarcopenia assessments are often based on less reliable surrogate markers, such as calf circumference, which can be strongly influenced by conditions like oedema, reducing diagnostic accuracy.

The primary objective of our study is to overcome these limitations by utilizing a more precise and reliable diagnostic approach for sarcopenia: US measurement of the quadriceps femoris muscle and its pennation angle. The aim of this paper is to accurately evaluate the correlation between sarcopenia and delirium in patients admitted to our Geriatrics Division—by analysing robust and specific data, we look to present findings that support the implementation of more targeted assessments and interventions for our high‐risk patient population.

## 2 Methods

### 2.1 Study Design and Participants

**FIGURE 1:**
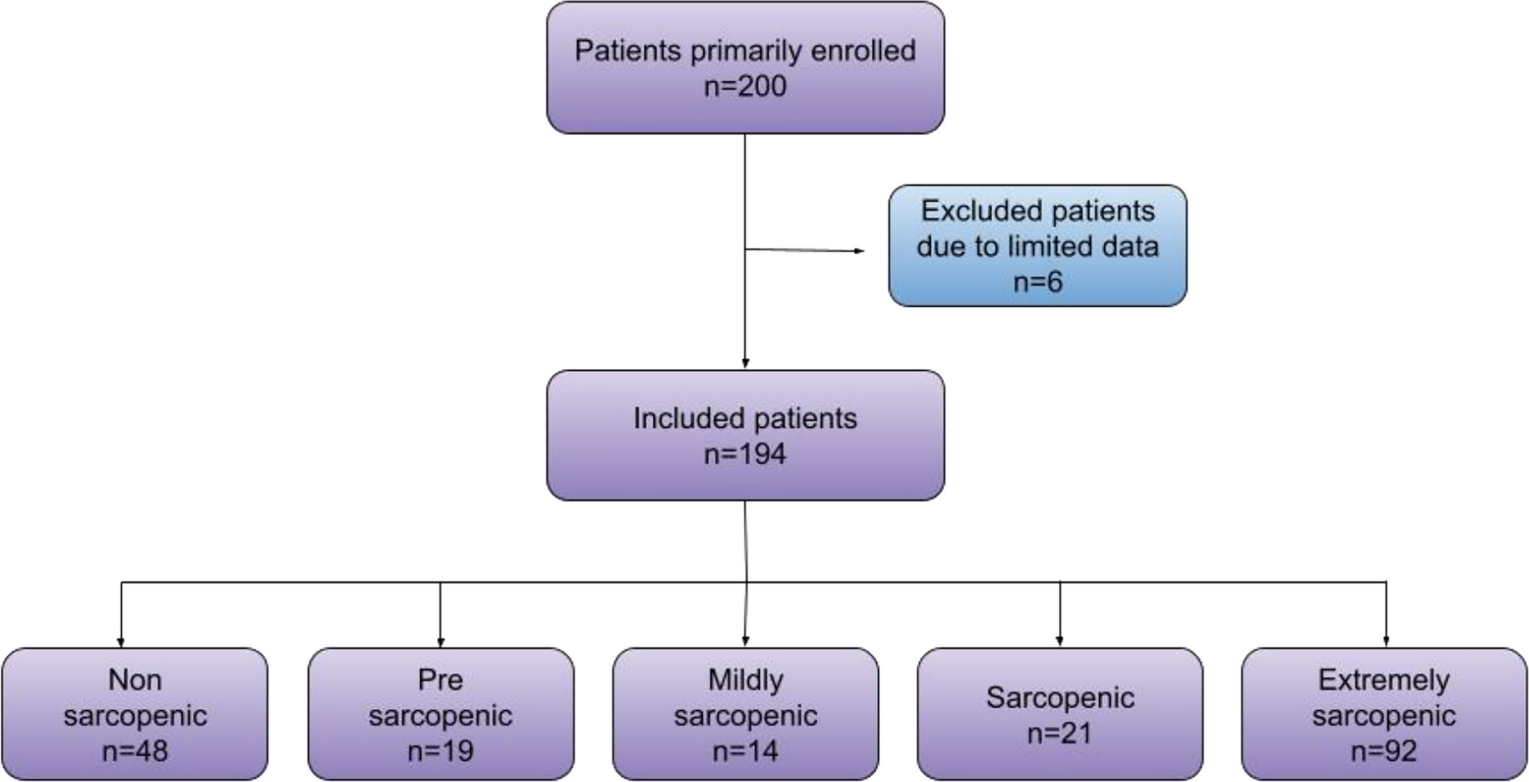
Flowchart of patient inclusion and posterior classification based on the sarcopenic level.

This prospective, single‐centre study was conducted at the Geriatric Division of San Luigi Gonzaga Hospital in Orbassano, Turin, Italy. Initial enrolment consisted of 200 patients aged ≥ 65 years who were admitted through the Emergency Department between 1 February 2025 and 31 July 2025. All participants remained hospitalized for more than 24 h. From an initial cohort of 200 patients, 194 patients (90 females, 104 males) were included in the final analysis. Six patients were excluded from the initial enrolment due to incomplete clinical or US data, resulting in a final cohort of 194 patients for analysis. The mean age was 86.5 years (range, 67–102 years). All hospitalized patients originated from the hospital's emergency department and remained in boarding for approximately 60 h prior to the transfer to the Geriatrics Department (Figure 1).

### 2.2 Data Collection

Upon admission, all patients underwent a comprehensive assessment on clinical, functional, cognitive and laboratory levels (Table 1). The average weight and height were 59.5 kg and 1.60 m, respectively, resulting in a mean BMI of 22.72—falling within the normal weight range.

**TABLE 1:** Baseline characteristics of the patients.

		Not sarcopenic	Pre‐sarcopenic	Mildly sarcopenic	Sarcopenic	Extremely sarcopenic	Total
Demographic	Patients, *n* (%)	48 (24.7)	19 (9.8)	14 (7.2)	21 (10.8)	92 (47.5)	194
	Male, *n* (%)	31 (64,5)	13 (68.4)	7 (50)	10 (47.6)	43 (46.7)	104 (53.6)
	Age (years)	84.35 ± 6.7	84.63 ± 6.9	86.42 ± 6.3	87.38 ± 6.3	87.82 ± 6.3	86.52 ± 6.6
	Weight (kg)	68.7 ± 15.3	61.8 ± 14.2	58 ± 12,6	57.1 ± 10.3	55.1 ± 12.4	59.5 ± 14.2
	Height (m)	1.62 ± 0.1	1.62 ± 0.1	1.59 ± 0.1	1.57 ± 0.09	1.6 ± 0.09	1.6 ± 0.09
Delirium							
	Patients, *n* (%)^a^	20 (41.6)	16 (84.2)	13 (92.8)	20 (95.2)	89 (96.7)	158 (81.4)
Cognitive							
	MMSE	24 (22–26)	22 (20–24)	21 (18–23)	19 (15–23)	13 (9–18)	19 (12–23)
Functional							
	ADL	3 (1.75–6)	1 (0–3)	1.5 (1–4.5)	1 (0–3)	1 (0–2)	1 (0–3)
	IADL	2 (0–4)	0 (0–2)	0.5 (0–2.8)	0 (0–1)	0 (0–1)	0 (0–2)
Muscle and strength							
	USSI	3.29 ± 0.8	4.43 ± 0.2	4.98 ± 0.1	5.59 ± 0.1	9.27 ± 3.2	6.61 ± 3.47
	HGS (kg)	20.4 ± 6.8	14.6 ± 3.8	15.7 ± 7.8	14.7 ± 5.5	8.6 ± 4.7	13.3 ± 7.4
	BMI (kg/m^2^)	26 ± 5,5	23,5 ± 4,2	22.9 ± 4.5	23.1 ± 3.3	20.0 ± 4.5	22.7 ± 6.3
	CFS	4 (2–7)	8 (5–8)	7.5 (4–8)	8 (6–8)	8 (7–9)	7 (4–8)
Markers							
	Na (mmol/L)	139.9 ± 4.1	140.4 ± 6.7	129.6 ± 3.05	137.7 ± 6.5	141.3 ± 5.6	140.3 ± 5.4
	Fe (μg/dL)	46 (34.5–65.5)	30 (25–50.5)	36 (31.5–69.2)	62 (23–91)	42.5 (26–64)	44 (28–65)
	Ferr (ng/mL)	292 (153–568.5)	211 (87–266)	234 (142.7–341.5)	238 (161–322)	306.5 (169.2–578.7)	283 (152–566)
	Tf (mg/dL)	180.2 ± 45.2	108.9 ± 53.2	172 ± 32.5	178.5 ± 69.8	167.6 ± 40.4	173.5 ± 46.3
	TC (mg/dL)	153.5 ± 45.5	137.9 ± 37.7	138.4 ± 34.5	143.9 ± 38.6	141.3 ± 40.8	144.0 ± 41.1
	TP (g/dL)	5.7 ± 0.6	5.6 ± 0.6	5.9 ± 0.8	5.3 ± 0.7	5.7 ± 1.6	5.7 ± 1.2
	TG (mg/dL)	113.5 ± 51.1	104.1 ± 61.6	118.6 ± 59.9	100.5 ± 48.5	104.7 ± 41.2	107.4 ± 47.9
	Folate (ng/mL)	4.3 (3–6.6)	4.6 (3.3–7.5)	9.6 (3.5–7.2)	4.1 (3.2–7.2)	4.4 (2.7–6.8)	4.4 (3.0–7.1)
	Vit D (ng/mL)	13.2 (8.2–26.1)	13.5 (8.3–18.8)	10.6 (4.7–15.5)	12.5 (7–21)	11.4 (6.5–22.4)	12.4 (6.8–23.1)
	Vit B12 (pg/mL)	393 (251–587)	299 (223–420)	523.5 (348–761)	434.5 (246–765)	361.5 (249.7–541.5)	374 (250.2–576)
	TSH (mcIU/mL)	1.2 (0.8–1.8)	0.9 (0.5–1.5)	0.6 (0.3–1)	1.3 (0.5–2.2)	1.0 (0.6–1.7)	1 (0.6–1.8)
	CRP (mg/dL)	3.2 (1.3–7.3)	4.1 (2–6.7)	3.3 (2.9–12.5)	3.0 (1.2–4.9)	4.1 (1.4–10.5)	3.7 (1.3–8.6)
	Alb (g/L)	29.4 ± 5.3	27.2 ± 5.3	29.7 ± 9.0	27.9 ± 7.4	27.2 ± 4.4	28.0 ± 5.5
	Prealb (mg/dL)	15.4 ± 6.7	14.7 ± 5.0	15 ± 7.2	13.3 ± 4.0	13 ± 4.8	14.0 ± 5.6

*Note:* Values are presented as mean ± standard deviation (SD) for normally distributed variables and as median (interquartile range [IQR]) for non‐normally distributed or ordinal variables.

Abbreviations: ADL, Activities of Daily Living (0–6); Alb, albumin; BMI, body mass index; CFS, Clinical Frailty Scale (1–9); CRP, C‐reactive protein; Fe, iron; Ferr, ferritin; HGS, handgrip strength; IADL, Instrumental Activities of Daily Living (0–8); MMSE, Mini‐Mental State Examination (0–30); Na, sodium; Prealb, prealbumin; TC, total cholesterol; Tf, transferrin; TG, triglycerides; TP, total proteins; TSH, thyroid‐stimulating hormone; USSI, Ultrasound Sarcopenia Score Index (unitless; categorized as non‐sarcopenic, pre‐sarcopenic, mild, moderate or extreme sarcopenia); Vit, vitamin.

^a^ Delirium was assessed using the 4AT: 4 A's Test for delirium (0–12).

In addition to patients' height and weight, routine blood tests were performed to evaluate a range of clinical markers. These included inflammatory markers (C‐reactive protein [CRP]), electrolytes (sodium), thyroid function (TSH), metabolic markers (total cholesterol and triglycerides), nutritional status indicators (albumin, pre‐albumin and total protein), iron metabolism markers (serum iron, ferritin and transferrin), and vitamin levels (vitamin B12, folate and vitamin D). The Charlson Comorbidity Index (CCI) was used to assess the burden of comorbidity.

Cognitive and functional status were evaluated using standardized scales and tests. Cognitive function was assessed with the Mini‐Mental State Examination (MMSE), whereas functional independence was measured using the Activities of Daily Living (ADL) and Instrumental Activities of Daily Living (IADL) scales.

Frailty was assessed using the Clinical Frailty Scale (CFS), whereas delirium was screened using the 4‐Point Assessment Test (4AT), a validated tool with high sensitivity and specificity, useful for identifying both hyperactive and hypoactive forms of delirium. The combination of the CFS and 4AT was chosen to provide a holistic view of delirium risk by integrating both physical health and cognitive fluctuations.

A certified clinical sonographer performed the USSI assessments by measuring the vastus lateralis muscle thickness and pennation angle. These measurements were obtained using an US machine with a 7.5‐MHz linear probe, based on a previously validated protocol. The US protocol for muscle parameter assessment was standardized and performed under controlled conditions. All patients were examined in the supine position with the lower limbs slightly flexed to ensure relaxation of the quadriceps musculature and to optimize acoustic access. A high‐frequency linear transducer was used, with the device set to the musculoskeletal/deep muscle preset to enhance visualization of fascial planes and muscle architecture. The probe was initially positioned at the level of the knee, over the quadriceps femoris muscle group, and subsequently adjusted to obtain optimal longitudinal and transverse images according to the validated methodological recommendations. Grip strength was measured using a Gripix digital dynamometer, with settings adjusted for each patient's age and sex.

## 3 Statistical Analysis

Statistical analyses were performed using Jamovi (Version 2.3; The Jamovi Project) and the R statistical programming language (Version 4.1; R Foundation for Statistical Computing). The normality of data distributions was assessed using the Shapiro–Wilk test and inspection of Q–Q plots. Correlation analyses were conducted using Pearson's correlation coefficient (*r*) for normally distributed variables or Spearman's rank correlation coefficient (ρ) for non‐normally distributed data, as appropriate.

To account for potential confounding, partial correlation analyses were performed controlling for age and CRP (as expression of inflammatory status). Associations between USSI and cognitive/functional measures remained significant and of similar magnitude after adjustment, indicating that these relationships were not explained by age or inflammatory status. Partial correlation analyses were preferred as this approach adequately accounts for the ordinal and non‐continuous nature of several clinical scales used in the study. More advanced multivariable regression models would have required statistical assumptions (e.g., linearity, homoscedasticity and distributional properties) that are difficult to satisfy with these functional and cognitive assessment scores. Partial correlations therefore provided an appropriate and statistically robust method to adjust for confounders without imposing constraints that the data could not reliably meet.

Comparisons between two independent groups were performed using the independent‐samples *t*‐test for parametric data or the Mann–Whitney *U* test for non‐parametric data. Multiple group comparisons for non‐parametric data were conducted using the Kruskal–Wallis test. A *p*‐value < 0.05 was considered statistically significant.

### 3.1 Ethical Considerations

The study was conducted in accordance with the principles of the Declaration of Helsinki. All participants or their legally authorized representatives provided written informed consent for enrollment in the study.

## 4 Results

A total of 194 patients were included in the final analysis, with a mean age of 86.5 years (range 67–102); 90 were women (46.4%) and 104 men (53.6%). The average BMI was 22.7, and the mean MMSE score was 18.06. A significant majority of the patients, 158 out of 194 (81.44%), were diagnosed with delirium.

Statistical analyses revealed a clear correlation between the USSI and several clinical functions. Regarding cognitive function and delirium risk, a moderate‐to‐high negative correlation was observed between USSI and the MMSE score. The Pearson correlation coefficient was *r* = −0.619 (95% CI, −0.699 to −0.523; *p* < 0.001), and the Spearman coefficient was ρ = −0.670 (*p* < 0.001; *N* = 194). This indicates that higher USSI values, suggesting more severe sarcopenia, are associated with lower MMSE scores, suggesting poorer cognitive function. Similarly, a very strong positive correlation was found with the 4AT for delirium, with a Pearson coefficient of *r* = 0.844 (95% CI, 0.797–0.880; *p* < 0.001) and a Spearman coefficient of ρ = 0.841 (*p* < 0.001; *N* = 194). This data demonstrates that an elevated USSI is strongly associated with a higher risk or greater severity of delirium.

**FIGURE 2:**
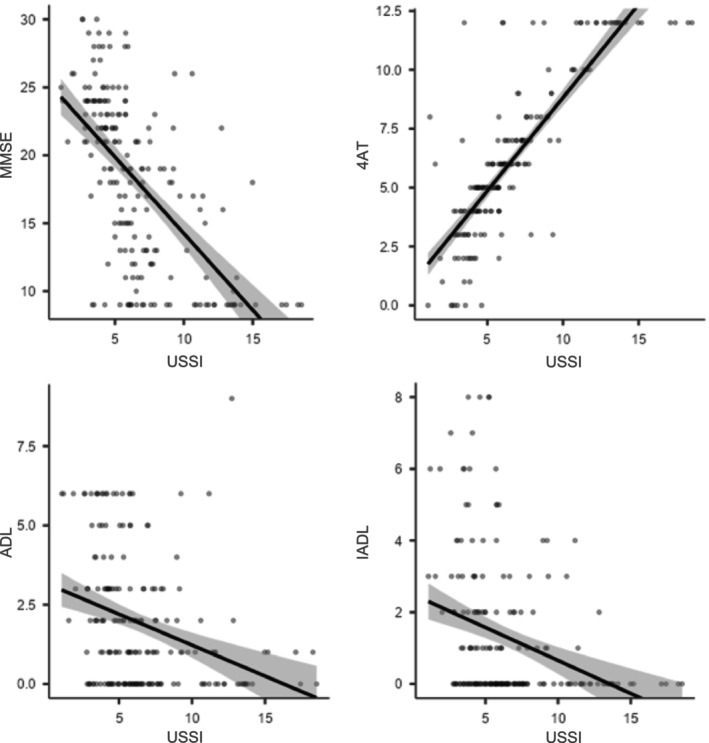
Correlation between the Ultrasound Sarcopenia Score Index (USSI) and cognitive/functional measures in older hospitalized patients. Scatter plots with fitted regression lines (solid black) and 95% confidence intervals (shaded areas) depict the relationships between USSI and Mini‐Mental State Examination (MMSE, top left; Spearman's *rho* = −0.670, 95% CI [−0.699, −0.523], *p* < 0.001), 4 A's Test (4AT, top right; Spearman's *rho* = 0.841, 95% CI [0.797, 0.880], *p* < 0.001), Activities of Daily Living (ADL, bottom left; Spearman's *rho* = −0.367, 95% CI [−0.445, −0.192], *p* < 0.001) and Instrumental Activities of Daily Living (IADL, bottom right; Spearman's *rho* = −0.375, 95% CI [−0.444, −0.191], *p* < 0.001). Higher USSI values were significantly associated with lower MMSE, ADL and IADL scores and with higher 4AT scores, indicating that greater sarcopenia severity is linked to poorer cognitive performance and reduced functional independence.

In the analysis of functional autonomy, a weak‐to‐moderate negative correlation was identified between USSI and the ADL and IADL scales. The Pearson coefficients for ADL and IADL were *r* = −0.324 (, −0.445 to −0.192; *p* < 0.001) and *r* = −0.323 (95% CI, −0.444; −0.191; *p* < 0.001), respectively, whereas the Spearman coefficients were ρ = −0.367 and ρ = −0.375. Although these correlations are less pronounced than those observed for cognitive function, they still indicate that a higher USSI is associated with reduced autonomy in daily life (Figure 2).

In conclusion, the statistical data support the clinical hypothesis that a higher USSI, and therefore more severe sarcopenia, is significantly correlated with poorer cognitive status, higher risk of delirium and reduced functional autonomy. The strongest associations were observed between muscle status (USSI) and neurological functions, highlighting the relationship between sarcopenia and cognitive health.

Further analysis revealed significant associations between the USSI and various nutritional and muscular markers. Conversely, weak negative associations were found between USSI and nutritional markers such as albumin, prealbumin and transferrin, suggesting that higher USSI values tend to occur in the context of protein–calorie malnutrition, consistent with sarcopenia. No significant correlations emerged with other markers, including ferritin, iron, cholesterol, folate, vitamins D and B12 or TSH, indicating that the relationship is specific to inflammation and nutrition markers.

The analysis also confirms the validity of the USSI by demonstrating strong and significant correlations with established muscle parameters. A strong negative association was found between USSI and body mass index (BMI), grip strength (DYN), and muscle thickness (TM), indicating that a higher USSI corresponds to lower values of BMI, strength and muscle mass and hence supports the hypothesis that a high USSI score is indicative of more severe sarcopenia. On the other hand, the fascial layer (LF) shows a strong positive association with USSI, a finding consistent with the typical structural changes of sarcopenia. Overall, these results indicate the USSI is a consistent and promising tool for the assessment of sarcopenia.

Sex‐based comparisons revealed several significant differences. Women had significantly lower stature, weight and body mass index (BMI) compared to men. Their muscle performance was also inferior, as evidenced by lower grip strength (DYN) and muscle thickness (TM), although no significant difference was observed in muscle fascicle length (LF). Regarding muscle quality, women exhibited higher USSI scores, indicating poorer muscle quality characterized by greater fibrosis and inflammation.

Regarding clinical and cognitive status, women displayed a less favourable profile: lower score on the MMSE, indicating reduced cognitive function, and higher score on the 4AT, indicating a greater risk of delirium. They also had higher scores on the CFS, suggesting increased frailty. However, no significant differences were found in ADL and IADL (Table 2).

**TABLE 2:** Comparative analysis between men and women.

	Women (*n* = 90)	Men (*n* = 104)	Δ (mean difference) [95% CI]	*p*	Effect size (*r* biserial)
Height (m)	1.55 ± 0.07	1.66 ± 0.08	−0.110 [−0.132, −0.088]	< 0.001	0.7
Weight (kg)	54 ± 13.1	64.3 ± 13.5	−10.31 [−14.09, −6.53]	< 0.001	0.45
BMI (kg/m^2^)	21.2 ± 6.4	23.7 ± 5.9	−2.75 [−4.51, −1.00]	0.002	0.25
HGS (kg)	11.2 ± 5.5	15.2 ± 8.2	−4.03 [−6.05, −2.01]	< 0.001	0.27
LF (mm)	48.1 ± 12.3	45.4 ± 10.5	NS	0.098	NS
TM (mm)	7.5 ± 2.9	8.9 ± 3.1	−1.39 [−2.24, −0.53]	0.002	0.27
USSI	7.55 ± 4.0	5.79 ± 2.6	1.75 [0.8, 2.71]	< 0.001	0.24
MMSE	17 (9–22.7)	21 (15–24)	−2.36 [−4.13, −0.60]	0.009	0.21
4AT	6 (4–8.7)	5 (4–7)	1.28 [0.38, 2.19]	0.006	0.22
CFS	8 (6–9)	7 (4–8)	0.75 [0.06, 1.44]	0.034	0.17
ADL	1 (0–2)	1.5 (0–3.2)	NS	0.087	NS
IADL	0 (0–2)	0 (0–2)	NS	0.511	NS

*Note:* Comparison of anthropometric, muscle and cognitive‐functional parameters between women and men. Values are presented as mean ± standard deviation (SD) for normally distributed variables, and as median (interquartile range [IQR]) for non‐normally distributed or ordinal variables.

Abbreviations: 4AT, 4 A's Test for delirium (0–12); ADL, Activities of Daily Living (0–6); BMI, body mass index; CFS, Clinical Frailty Scale (1–9); DYN, dynamometer; IADL, Instrumental Activities of Daily Living (0–8); LF, muscle fascicle length; MMSE, Mini‐Mental State Examination (0–30); NS, not significant; TM, muscle thickness; USSI, Ultrasound Sarcopenia Severity Index.

**FIGURE 3:**
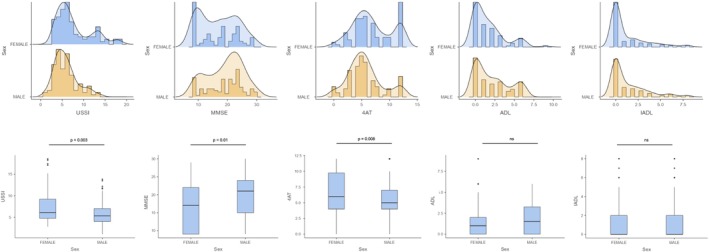
Panel comparing females versus males for ultrasound‐derived sarcopenia and cognitive/functional performance (top row: histograms with density line, bottom row: boxplots). Females presented a higher Ultrasound Sarcopenia Score Index (USSI) than males (7.55 ± 4.01 vs. 5.79 ± 2.68; *p* = 0.003), lower Mini‐Mental State Examination (MMSE) scores (16.80 ± 6.46 vs. 19.16 ± 6.00; *p* = 0.010) and higher 4 A's Test (4AT) scores (6.82 ± 3.40 vs. 5.54 ± 3.00; *p* = 0.008). By contrast, Activities of Daily Living (ADL) and Instrumental ADL (IADL) did not differ significantly between sexes (ADL: 1.61 ± 1.98 vs. 2.13 ± 2.16, *p* = 0.108; IADL: 1.18 ± 1.99 vs. 1.37 ± 1.97, *p* = 0.239). Overall, females showed a greater ultrasound‐estimated sarcopenia and worse cognitive scores, with similar functional dependence across sexes.

Correlational analyses between USSI, DYN and various clinical and functional outcomes in both sexes further demonstrated that higher USSI scores, indicative of poorer muscle quality, were associated with less favourable clinical profiles, including higher 4AT scores and lower MMSE, ADL and IADL scores, in both sexes. In men, a higher USSI was additionally correlated with lower TM, higher LF and reduced DYN. In general, lower grip strength (DYN) was associated with a poorer cognitive and functional status, with lower scores on MMSE, ADL and IADL and higher scores on 4AT and CFS, indicating greater frailty. Moreover, in both sexes, reduced muscle thickness (TM) was associated with lower MMSE scores (Figure 3).

Finally, it is noteworthy that the analysis did not detect significant differences in age, strength (DYN), muscle thickness (TM), muscle quality (USSI), BMI or clinical outcomes (MMSE, ADL, IADL, 4AT and CFS) across different sarcopenia severity grades (Table 3). The lack of significant differences is likely attributable to the high degree of homogeneity in our cohort, which consisted of acutely ill, hospitalized geriatric patients with advanced age and a high baseline level of frailty. This narrow range of severe morbidity likely created a ‘ceiling effect’, minimizing detectable differences between the predefined severity groups. This suggests that, within the context of the presented study, sarcopenia classification did not provide a clear and meaningful distinction for these specific parameters.

**TABLE 3:** Correlation analysis stratified by sex.

		Women *r* (*p*)	Men *r* (*p*)
USSI	4AT	0.878 (< 0.001)	0.796 (< 0.001)
	MMSE	−0.573 (< 0.001)	−0.660 (< 0.001)
	ADL	−0.284 (< 0.01)	−0.350 (< 0.001)
	IADL	−0.364 (< 0.001)	−0.282 (< 0.01)
	DYN	NS	−0.601 (< 0.001)
DYN			
	4AT	−0.819 (< 0.001)	−0.536 (< 0.001)
	MMSE	0.613 (< 0.001)	0.567 (< 0.001)
	ADL	0.375 (< 0.001)	0.492 (< 0.001)
	IADL	0.465 (< 0.001)	0.491 (< 0.001)
	CFS	−0.450 (< 0.001)	−0.546 (< 0.001)
TM			
	MMSE	0.575 (< 0.001)	NS

Abbreviations: 4AT, 4 A's Test; ADL, Activities of Daily Living; CFS, Clinical Frailty Scale; DYN, handgrip strength; IADL, Instrumental Activities of Daily Living; MMSE, Mini‐Mental State Examination; NS, not significant; r, Spearman's correlation coefficient; USSI, Ultrasound Sarcopenia Score Index.

## 5 Discussion

The USSI is a recently validated US‐based tool designed to assess muscle mass [17]. It is calculated as the ratio between the thickness of the vastus lateralis muscle and its fascicle length. This index allows for both a quantitative and qualitative evaluation of muscle quality and mass, independently of the patient's sex and body size [17]. The USSI represents a promising alternative to traditional diagnostic methods like Magnetic Resonance Imaging (MRI) and Dual‐Energy X‐ray Absorptiometry (DXA) [27].

The USSI offers several advantages, particularly for the assessment of frail, elderly patients. It can be performed at the patient's bedside, is rapid, and, unlike DXA, does not involve exposure to ionizing radiation [27, 28]. Furthermore, the USSI is not contraindicated in the presence of prosthetic devices, metallic implants, or pacemakers, which can be a limitation for MRI [29]. Its independence from sex and body size further enhances its applicability across a broad range of patients [17, 27].

However, the USSI also has limitations. Its accuracy may be reduced in patients with severe obesity or anatomical abnormalities that impair adequate muscle visualization [30]. Specialized training is required for image acquisition and interpretation. In cases where muscle fascicles are particularly long, they may not be fully visible, necessitating external software for more precise measurements.

Our study explored the correlations between the USSI and various clinical conditions, yielding significant findings. A strong inverse correlation was found between the USI value and the 4AT scale, a validated tool for delirium screening. Notably, the study reported a very high incidence of delirium (81.44%), likely due to the patients' prolonged stay in the emergency department, an environment known to precipitate this condition (Fraccalini et al.) [19], as well as the high mean age noted (86.5 years). It is important to note that factors such as agitation, disorientation, and fever, common in septic patients enrolled in the study, may have negatively influenced patient cooperation and, consequently, the results of cognitive tests like the 4AT or the Mini‐Mental State Examination (MMSE).

A significant, albeit weak, correlation was also observed between the USSI and the functional status of patients, as measured by the Activities of Daily Living (ADL) and Instrumental Activities of Daily Living (IADL) scales [31]. This finding supports the hypothesis that sarcopenia limits a patient's physical function, affecting their ability to perform both self‐care and instrumental daily activities [31, 32].

Interestingly, a moderate positive correlation (r = 0.282, *p* < 0.001) was found between the USSI and inflammatory markers, specifically C‐reactive protein (CRP) levels. This result is consistent with the theory that sarcopenia is associated with a systemic inflammation component, commonly known as “inflammaging,” which can act as an etiopathogenic cofactor [33, 34].

It is also important to consider the effect sex in sarcopenia holds. Our cohort saw women exhibiting higher USSI scores indicative of poorer muscle quality, alongside worse cognitive and frailty scores. This finding reflects wider research suggesting that sarcopenia in women is strongly linked to nutritional status and a relative decline in anabolic hormones like Insulin‐like Growth Factor‐1 (IGF‐1) [35]. Furthermore, recent evidence confirms that this female vulnerability is evident at hospital admission, with studies in rehabilitation settings showing a significantly higher prevalence of sarcopenia in women, highlighting them as a key at‐risk group upon hospitalization [36].

Finally, only minor correlations were found between USSI and nutritional indices. This is likely because the cross‐sectional design of our study cannot establish causality; it remains unclear whether chronic malnutrition leads to sarcopenia or if the acute catabolic state of hospitalization simultaneously depresses both nutritional markers and muscle quality. Furthermore, the acute inflammatory state common in hospitalized elderly can independently depress serum albumin and prealbumin, confounding their interpretation. Our single time‐point measurement may not capture the chronic nutritional status required to significantly impact muscle architecture, and the sample size may have been insufficient to detect a more nuanced relationship. Furthermore, the homogeneity of our severely frail cohort may also explain the lack of significant differences across sarcopenia severity grades, as most patients presented at a similarly advanced stage of muscular and functional decline.

Despite the study's limitations, the results are promising. US‐based assessment of sarcopenia is an exceptional tool that can be performed quickly at the patient's bedside [37]. Our findings suggest that the USSI could serve as a predictive marker for delirium, facilitating the identification of frail, sarcopenic patients who are at a higher risk of developing delirium. The USSI may represent a pivotal advancement in the diagnosis and management of sarcopenia by providing fast and non‐invasive means for its early identification and longitudinal monitoring.

## 6 Conclusions

This study validates the USSI as a rapid, non‐invasive tool for assessing muscle mass and predicting clinical outcomes. Lower USSI values, indicative of sarcopenia, were strongly associated with increased risk of delirium, particularly in frail, hospitalized elderly patients. This association is particularly relevant for these patients, where delirium is common and linked to adverse clinical outcomes. Significant correlations with ADL and IADL scales further suggest a negative impact of sarcopenia on functional independence and quality of life. Additionally, the association between USSI and C‐reactive protein levels reinforces the role of systemic inflammation (‘inflammaging’) in sarcopenia's pathogenesis. Despite certain limitations, the USSI shows promise for early identification of at‐risk patients and timely therapeutic intervention.

Despite certain limitations, such as the potential influence of external factors (e.g., patient agitation) and the need for a larger sample size to validate nutritional data, our results suggest that the USSI holds substantial promise for transforming the diagnostic approach to sarcopenia. Early identification of at‐risk individuals via USSI may enable more effective monitoring and timely implementation of therapeutic strategies aimed at preventing or mitigating both sarcopenia and delirium.

## Funding

The authors have nothing to report.

## Conflicts of Interest

The authors declare no conflicts of interest.

## References

[1] Y. Shen , Q. Wan , R. Zhao , et al., “Low Skeletal Muscle Mass and the Incidence of Delirium in Hospitalized Older Patients: A Systematic Review and Meta‐Analysis of Observational Studies,” International Journal of Clinical Practice 2023 (2023): 4098212, 10.1155/2023/4098212. 37188154 PMC10181906

[2] K. Shiozaki , A. Nagano , M. Hanaoka , Y. Uchiyama , K. Domen , and T. Koyama , “The Association Between Possible Sarcopenia and Delirium Onset in Older Patients With Acute Stroke,” Cureus 17, no. 6 (2025): e86993, 10.7759/cureus.86993. 40589442 PMC12206565

[3] A. D. Ardeljan and R. Hurezeanu , “Sarcopenia,” in StatPearls (StatPearls Publishing, 2025). 32809648

[4] Y. Nakano , Y. Hirata , T. Shimogawara , et al., “Frailty Is a Useful Predictive Marker of Postoperative Complications After Pancreaticoduodenectomy,” World Journal of Surgical Oncology 18 (2020): 194, 10.1186/s12957-020-01969-7. 32746840 PMC7401197

[5] R. Vaishya and A. Vaish , “Falls in Older Adults Are Serious,” Indian Journal of Orthopaedics 54, no. 1 (2020): 69–74, 10.1007/s43465-019-00037-x. 32257019 PMC7093636

[6] S. E. Chou , C. S. Rau , Y. C. Tsai , S. Y. Hsu , H. Y. Hsieh , and C. H. Hsieh , “Risk Factors and Complications Contributing to Mortality in Elderly Patients With Fall‐Induced Femoral Fracture: A Cross‐Sectional Analysis Based on Trauma Registry Data of 2,407 Patients,” International Journal of Surgery 66 (2019): 48–52, 10.1016/j.ijsu.2019.04.010. 31026517

[7] A. J. Cruz‐Jentoft , J. P. Baeyens , J. M. Bauer , et al., “Sarcopenia: European Consensus on Definition and Diagnosis,” Age and Ageing 39, no. 4 (2010): 412–423, 10.1093/ageing/afq034. 20392703 PMC2886201

[8] J. D. Walston , “Sarcopenia in Older Adults,” Current Opinion in Rheumatology 24, no. 6 (2012): 623–627, 10.1097/BOR.0b013e328358d59b. 22955023 PMC4066461

[9] J. Xu , E. M. Reijnierse , J. Pacifico , C. S. Wan , and A. B. Maier , “Sarcopenia Is Associated With 3‐Month and 1‐Year Mortality in Geriatric Rehabilitation Inpatients: RESORT,” Age and Ageing 50, no. 6 (2021): 2147–2156, 10.1093/ageing/afab134. 34260683 PMC8581377

[10] W. T. Park , O. J. Shon , and G. B. Kim , “Multidisciplinary Approach to Sarcopenia: A Narrative Review,” Journal of Yeungnam Medical Science 40, no. 4 (2023): 352–363, 10.12701/jyms.2023.00724. 37674374 PMC10626311

[11] W. Wu , M. Liu , Q. Zeng , C. Tang , and J. Huo , “Research Progress on Evaluation Methods for Skeletal Muscle Mass Assessment in Sarcopenia (Review),” Oncology Letters 30, no. 3 (2025): 423, 10.3892/ol.2025.15169. 40688583 PMC12273789

[12] M. G. Borda , G. Duque , M. U. Pérez‐Zepeda , et al., “Using Magnetic Resonance Imaging to Measure Head Muscles: An Innovative Method to Opportunistically Determine Muscle Mass and Detect Sarcopenia,” Journal of Cachexia, Sarcopenia and Muscle 15, no. 1 (2023): 189–197, 10.1002/jcsm.13362. 38050325 PMC10834349

[13] “Body Composition With Dual Energy X‐Ray Absorptiometry: From Basics to New Tools,” Messina – Quantitative Imaging in Medicine and Surgery,” accessed August 13, 2025, https://qims.amegroups.org/article/view/41830/html. 10.21037/qims.2020.03.02 PMC7378094 32742961

[14] T. Rondaij , N. R. Kozjek , C. Jerele , and T. Jordan , “Is There a Place for Ultrasound in Diagnosing Sarcopenia?,” Radiology and Oncology 59, no. 2 (2025): 153–167, 10.2478/raon-2025-0035. 40544501 PMC12182947

[15] L. Di Lenarda , A. Buoite Stella , C. Ratti , et al., “Assessing Muscle Mass in the Orthopedic Clinical Setting: Application of the Ultrasound Sarcopenia Index in Elderly Subjects With a Recent Femoral Fracture,” Nutrients 16, no. 5 (2024): 711, 10.3390/nu16050711. 38474844 PMC10934151

[16] S. Perkisas , S. Baudry , J. Bauer , et al., “Application of Ultrasound for Muscle Assessment in Sarcopenia: Towards Standardized Measurements,” European Geriatric Medicine 9, no. 6 (2018): 739–757, 10.1007/s41999-018-0104-9. 34674473

[17] M. Narici , J. McPhee , M. Conte , et al., “Age‐Related Alterations in Muscle Architecture Are a Signature of Sarcopenia: The Ultrasound Sarcopenia Index,” Journal of Cachexia, Sarcopenia and Muscle 12, no. 4 (2021): 973–982, 10.1002/jcsm.12720. 34060717 PMC8350200

[18] F. Thomas , R. Valerio , T. Beatrice , et al., “Study on the Occurrence of Delirium in Geriatric Patients Undergoing Hip Fracture Surgery,” Minerva Psychiatry 65, no. 2 (2024): 163–169.

[19] T. Fraccalini , A. Trogolo , M. Traversa , et al., “Delirium in the Emergency Department: Incidence and Risk Factors in a Ligurian Hospital,” Archives of Gerontology and Geriatrics Plus 2, no. 2 (2025): 100165, 10.1016/j.aggp.2025.100165.

[20] T. Fraccalini , I. R. Bergoglio , G. Fonte , et al., “Lung Ultrasound (LUS) as a Predictor of Delirium in Elderly Patients With COVID‐19 During Hospitalization: A Geriatric Vulnerability‐Stress Model,” Archives of Gerontology and Geriatrics Plus 2, no. 3 (2025): 100186, 10.1016/j.aggp.2025.100186.

[21] F. Thomas , “Delirium and COVID‐19: Prevalence, Outcomes and Associated Factors in a Cohort of Elderly Inpatients,” Minerva Psychiatry 66, no. 2 (2025): 100–107.

[22] C. Huang , B. Wu , H. Chen , et al., “Delirium in Psychiatric Settings: Risk Factors and Assessment Tools in Patients With Psychiatric Illness: A Scoping Review,” BMC Nursing 23 (2024): 464, 10.1186/s12912-024-02121-6. 38977984 PMC11229275

[23] X. Mei , Y. H. Liu , Y. Q. Han , and C. Y. Zheng , “Risk Factors, Preventive Interventions, Overlapping Symptoms, and Clinical Measures of Delirium in Elderly Patients,” World Journal of Psychiatry 13, no. 12 (2023): 973–984, 10.5498/wjp.v13.i12.973. 38186721 PMC10768493

[24] M. J. M. M. van der Steen‐Dieperink , W. A. C. Koekkoek , and I. W. K. Kouw , “Sarcopenia and Frailty in Critical Illness,” Current Opinion in Clinical Nutrition and Metabolic Care 28, no. 3 (2025): 192–199, 10.1097/MCO.0000000000001123. 40072495 PMC11970596

[25] G. Bellelli , F. Triolo , M. C. Ferrara , et al., “Delirium and Frailty in Older Adults: Clinical Overlap and Biological Underpinnings,” Journal of Internal Medicine 296, no. 5 (2024): 382–398, 10.1111/joim.20014. 39352688

[26] G. Bellelli , A. Zambon , S. Volpato , et al., “The Association Between Delirium and Sarcopenia in Older Adult Patients Admitted to Acute Geriatrics Units: Results From the GLISTEN Multicenter Observational Study,” Clinical Nutrition (Edinburgh, Scotland) 37, no. 5 (2018): 1498–1504, 10.1016/j.clnu.2017.08.027. 28918171

[27] A. Ticinesi , T. Meschi , M. V. Narici , F. Lauretani , and M. Maggio , “Muscle Ultrasound and Sarcopenia in Older Individuals: A Clinical Perspective,” Journal of the American Medical Directors Association 18, no. 4 (2017): 290–300, 10.1016/j.jamda.2016.11.013. 28202349

[28] C. Messina , G. Maffi , J. A. Vitale , F. M. Ulivieri , G. Guglielmi , and L. M. Sconfienza , “Diagnostic Imaging of Osteoporosis and Sarcopenia: A Narrative Review,” Quantitative Imaging in Medicine and Surgery 8, no. 1 (2018): 86–99, 10.21037/qims.2018.01.01. 29541625 PMC5835659

[29] M. Ghadimi and A. Thomas , “Magnetic Resonance Imaging Contraindications,” in StatPearls (StatPearls Publishing, 2025). 31869133

[30] L. M. Donini , L. Busetto , S. C. Bischoff , et al., “Definition and Diagnostic Criteria for Sarcopenic Obesity: ESPEN and EASO Consensus Statement,” Obesity Facts 15, no. 3 (2022): 321–335, 10.1159/000521241. 35196654 PMC9210010

[31] H. Zhou , X. Ding , and M. Luo , “The Association Between Sarcopenia and Functional Disability in Older Adults,” Journal of Nutrition, Health & Aging 28, no. 1 (2024): 100016, 10.1016/j.jnha.2023.100016.38267154

[32] Y. Rolland , S. Czerwinski , G. A. Van Kan , et al., “Sarcopenia: Its Assessment, Etiology, Pathogenesis, Consequences and Future Perspectives,” Journal of Nutrition, Health & Aging 12, no. 7 (2008): 433–450, 10.1007/BF02982704. PMC3988678 18615225

[33] E. Antuña , C. Cachán‐Vega , J. C. Bermejo‐Millo , et al., “Inflammaging: Implications in Sarcopenia,” International Journal of Molecular Sciences 23, no. 23 (2022): 15039, 10.3390/ijms232315039. 36499366 PMC9740553

[34] S. Dalle , L. Rossmeislova , and K. Koppo , “The Role of Inflammation in Age‐Related Sarcopenia,” Frontiers in Physiology 8 (2017): 1045, 10.3389/fphys.2017.01045. 29311975 PMC5733049

[35] L. Tay , Y. Y. Ding , B. P. Leung , et al., “Sex‐Specific Differences in Risk Factors for Sarcopenia Amongst Community‐Dwelling Older Adults,” Age (Dordrecht, Netherlands) 37, no. 6 (2015): 121, 10.1007/s11357-015-9860-3. 26607157 PMC5005859

[36] Y. Yoshimura , H. Wakabayashi , F. Nagano , et al., “Sex Differences in Sarcopenia Prevalence and Muscle‐Related Outcomes Among Post‐Stroke Inpatients,” European Geriatric Medicine 16 (2025): 1071–1079, 10.1007/s41999-025-01186-z. 40123027

[37] G. Esposto , R. Borriello , L. Galasso , et al., “Ultrasound Evaluation of Sarcopenia in Patients With Hepatocellular Carcinoma: A Faster and Easier Way to Detect Patients at Risk,” Diagnostics 14, no. 4 (2024): 371, 10.3390/diagnostics14040371. 38396410 PMC10887735

